# Selections

**Published:** 1817-01

**Authors:** 


### PART III.

### SELECTIONS.

—— Factorum est copia nobis.

### I. Physiology.—*Memoir on the first Inspiration of the New-born Child.**

Respiration begins and terminates with life. Inspiration is the first phænomenon observed in the new-born child. Expiration follows: and these movements succeed each other regularly, and without interruption, until death; which always takes place after an expiration: so that to die and to expire are synonymous terms.

This important function has not failed to attract the attention of the philosophers of every age and country. Its mechanism, causes, effects, and relations with the other animal functions, have been successively investigated. Numerous observations and experiments were necessary to bring the physiological doctrine of respiration to the point which it has already attained: and, notwithstanding these labours, much obscurity remains to be cleared up; many questions to be subjected to a new and impartial examination.

Of these questions, one of the most obscure and important is that which regards the causes of the first inspiration. What incites the infant to dilate its thorax, and seek in a new element the means of supporting life? This query, so often proposed by physiologists, no one has yet satisfactorily resolved. By a review of the various opinions which have prevailed on this subject, we shall better expose the state of the question, and at the same time institute a point of departure for our own researches.

The cause of the first inspiration was referred by the ancients to the peculiar faculty possessed by the lungs, of absorbing atmospheric air,†—a faculty now refused, by physiologists, to the pulmonary organ; which is universally allowed to be wholly passive in inspiration, and governed by the movements of the thorax. On this passive state of the lungs, Baglivi,‡ and Pitcairne,§ have

---

* Mémoire sur la premiere inspiration de l'enfant nouveau né. Par Jean-Frederick Lobstein. Bulletin de la Société d'Emulation. No. iv. Avril. 1816.

† Gehler, De primâ fœtûs respiratione. Lipsiæ. 1777.

‡ Dissert. quarta de sang. et respirat. in oper. med. pract. Edit. nova. Lugd. 1733.

§ Dissert. de caus. div. mot. quâ sanguis fluit per pulmones. Sect. 14.

partly founded their explanation of the phænomenon in question; considering as its cause the tendency of the atmospheric air to preserve an equilibrium. The mouth of the new-born child, they contended, is commonly open; and hence the air, rushing into the lungs, forces the thorax to dilate.* But if the first respiration were merely executed according to the laws of aërostatics, nothing would prevent the fœtus from respiring even in a state of asphyxia; provided the mouth were kept open, and the larynx freed from obstructing mucus. According to this doctrine, it would even be possible to produce a sort of inspiration in a still-born child, if free access of air into the lungs were alone necessary.

Velthusius,† and Whytt,‡ suppose that the fœtus, while contained in the ovum, has a want of respiration, as imperious as the appetite for food at a later period; and which, suppressed during the placental connection of the fœtus with the mother, breaks out as soon as this connection is destroyed. The fœtus, then no longer receiving blood, its *pabulum vitæ*, from the mother, absorbs aliment from the atmosphere in which it is immersed. Whytt almost admits, in this appetite for respiration, a marked instinct on the part of the fœtus, and even an obscure evidence of volition. Setting aside the uncertainty of this conjectural doctrine, which, however, has been defended by Profesor Gehler, of Leipsic,§ how happens it that many new-born infants respire while yet connected with the mother, and supplied with vital aliment by the undivided umbilical cord?

From this consideration, Watt ‖ had already been induced to reject the doctrine of the other English physiologist, and admit, as the cause of the first inspiration, in the fœtus, a *desiderium vaginandi*, a want of expressing by cries the pain resulting from the compression which it has just suffered, and from exposure to the cold air. Haller ¶ entertained an opinion nearly similar; but can we, in sound physiology, infer that a function, so essential and important, depends on a circumstance, which, although frequent, does not invariably take place? Thus, for example, the respiratory function is executed by children, born in a state of apoplexy, while the life of relation yet continues depressed.

According to Diembroeck,** the heat, felt by the fœtus, and

---

* Baglivi says: Motus thoracis ab inflatis aëre pulmonibus pendet. And Pitcairne thus expresses himself: Irrumpit ac vi elateris et gravitatis, non autem dilitati prius pectoris compulsus.

† Tract. de Gener. c. 2. In Lamzweerde Swammerd. Respirat. expirat.

‡ Essay on the involuntary motions of animals.

§ Programma de fœtûs prima respiratione. Lips. 1773.

‖ Reflections on slow and painful labours and other subjects of midwifery.

¶ Prælect. in H. Boerhaav. propr. Institut. Tom. 5.

** Anatomes; L. 1. De Ventre infimo. cap. 34. Opera omnia. Genev. 4to. 1687.

which he supposes to augment towards the close of pregnancy, acts as a stimulus exciting the child to quit its prison, and refresh itself by respiration. This explanation, as attributing the exercise of volition to the inta-uterine fœtus, is exposed to the same objections which have been advanced against the preceding theories.

Already previously to Haller, it had been perceived that, in order to solve the problem of respiration, a first cause must be discovered, capable of exciting the inspiratory muscles.

Truston* thought that he had discovered this cause in the retention of the meconium, as well as in a *lucta sanguinis*, or effervescence of the blood, determined by the motions of the infant during delivery. Swammerdam† supposed the blood to be loaded with acrid humours, capable of producing in the chest a heat and effervescence which would stimulate the heart, the phrenic nerves, and respiratory muscles. He, moreover, taught that, in the fœtus yet contained within the ovum, the muscles of inspiration are in permanent action; endeavouring to overcome the resistance opposed to them by the membranes of the ovum and liquor amnii; but that, when the membranes have been ruptured, these muscles are no longer prevented from extending the thorax to its utmost limit of dilatation. According to Hamberger,‡ the first contraction of the respiratory muscles is an effect of the distention which their fibres experience during the passage of the child from the pelvis. In natural delivery, says this physiologist,§ the external intercostal muscles and diaphragm are tense, which state acts on these muscles as a stimulus inciting them to contract. When, on the other hand, the child comes by the feet, the internal intercostals are rendered tense: they contract and diminish the thorax; and, in this case, the life of the fœtus commences by an expiration, if, perchance, air, or other fluid, be contained in the pulmonary vesicles. Excepting the error which Hamberger has committed relative to the diversity of function of the internal and external intercostals, his opinion, as will be seen in the close of this memoir, is entitled to great attention.

Buffon|| made the first inspiration to depend on the action of the air upon the olfactory nerves and organs of respiration. The air, operating on these parts as a new stimulus, produces in them a kind of shock by which the ribs are raised, and its introduction into the lungs facilitated. However incontestible it be that the atmospheric fluid is to the fœtus a new excitant, wholly differing in impression from that which the liquor amnii exercises upon its surface, it is difficult to conceive why the air should act exclusively on the nerves of smell, and provoke sneezing. And even were this phænomenon invariably present, it is certain, that in order for

---

* De Respirat. usu primario. Londini, 1670.

† De Respirat. et usu pulmonum. Lud. Batav. 1667.

‡ Physiologia Medica. § Opus citatum.

|| Histoire naturelle de l'homme. Edit. in 4to. Tom. 2.

the air to strike the pituitary membrane, it must be first attracted by the nostrils. Now this attraction supposes a previous inspiration. Let any one present to his nostrils the most powerful odorous substance; but, without inspiration, the organ of smell will not be excited, nor the sensorium be apprized of the presence of the substance, however volatile. Thus, the opinion of Buffon, resting on a doubtful fact, affords no satisfactory explanation of the phænomenon in question.

Without investigating the cause of the first inspiration, Roederer* has indicated precisely the phænomena which accompany, and succeed, the birth of the child, and established the principle: 1st. That the fœtus cannot inspire till the whole of its thorax is expelled from the vulva; and 2dly. That the dilatation of the chest invariably precedes the first inspiration. Wrisberg† confirms these observations; and, moreover, remarks, that the thoracic muscles occasionally contract a certain time before the air descends into the lungs—a sure proof that the motions of the chest are always anterior to inspiration, and in no way determined by irruption of the aërial fluid into the pulmonary cells. This author attributes the first inspiration to the state of compression endured by the fœtus in the organs of the mother; and which causes contraction of most of the muscles, while the infant itself is excited to motion by a kind of instinct. And why, observes this physiologist, should we seek in accessory and external circumstances, the cause of the first inspiration: when the fœtus is already capable of sensible motion long before birth; and, consequently, habituated to the exercise of its muscular organs?‡ This opinion, while it limits the question, does not altogether resolve the difficulty. Granting that the motions of the fœtus, towards the close of pregnancy, depend chiefly on its muscles, there exists no proof that the diaphragm and intercostals contribute to their production. To admit the action of parts belonging to a function not yet established, would be as unsafe as to contend for the exercise of the organs of sense and digestion in the unborn fœtus. No system of fœtal organs possesses the power of entering into action, except it act effectively. It is true, that the possibility of respiration in the intra-ovine fœtus has lately been re-admitted, and the doctrine of Sennertus,§ Charleton,|| Tozzius,¶ and Manzinus,** thus re-intro-

---

* Satura de Suffocatis. Opuscula Medica. Goetting. tom. i.

† Programma de Respiratione primâ, nerv. phren. et calore animali. Goetting.

‡ Non opus sane est tam anxiè primam ad contrahendum expandendumque thoracem investigare rationem, in exteris quibusdam causis fœtum respicientibus, cum a primis, jam inde in utero materno temporibus matres ipsæ, ut plurimum embryonis inclusi testificentur motum.

§ Praxis Medica. Lib 4, Part ii, Sect 6.

|| De Respirat. Fœtus in Utero exercit. Phys. Anat. de Œconom. Anim. Lugd. Batav. 1678.

¶ Medic. pars prior Theoret. Lugd. 1681. Caput de Fœtûs Respiratione.

** Tract. de Fœtûs Respirat. Oper. tom. ii.

duced, by Professor Winslow, of Copenhagen, and other Philosophers of that city, who declare that they have seen the fœtus of several mammalia respire in the liquor amnii, while viewing these animals through the transparent membranes of the ovum.* Beclard,† has confirmed this extraordinary observation by establishing, as a positive fact, that the mechanical phænomena of respiration already exist in the fœtus. Granting that respiration may take place previously to birth, how are those cases to be explained in which the liquor amnii escapes some weeks before delivery, and leaves the fœtus absolutely dry in the interior of the ovum; notwithstanding which, it is often born alive and healthy? Deprived of the respirable fluid before birth, must it not cease to respire? Must not the respiratory organs, so long doomed to inactivity, lose the habit of acting? And will not the infant, when born, have to learn respiration anew, as though it had never exercised this function?

Dr. Bostock,‡ in fine, considers the first inspiration as the effect of the great dilatation of the chest, consequent on cessation of the pressure experienced by it during pregnancy and parturition. This explanation, in which the laws of vitality are forgotten, reposes too exclusively on mechanical principles, to be admitted without restriction: although, like that of Hamberger, it seems to contain somewhat of truth.

From this short historical notice, we may conclude, that physiologists are far from being unanimous respecting the cause of the first inspiration; and that this cause is still unknown. Yet Hamberger, we shall presently prove, had caught a glimpse of it; but, either from prejudice or want of the necessary development, it has been forgotten, and never received general assent.

Let us, ere we proceed, recollect that the diaphragm is the principal agent in natural respiration; that, on contraction, it descends in the abdominal cavity, and hence dilates the capacity of the thorax, and consequently that of the lungs, from whence results a space in the pulmonary vesicles, filled with rarified air; into which the external air, by virtue of its tendency to preserve an equilibrium, immediately rushes; but that the diaphragm, on relaxing, re-ascends towards the chest, diminishes its capacity, and of course that of the lungs, expels a portion of the contained air, and thus produces expiration. The question, therefore, respecting the causes of the first inspiration, naturally reduces itself to this: What are the causes which determine the first contraction of the diaphragm? To this question we are about to reply.

---

* Paul Scheel, Ueber Beschaffenheit und nutzen des Frucht-wasser in der Luftrohre der menschlichen Früchte. A. D. Lotaix. Erlang. 1800

† Chef des travaux Anatomiques à la Faculté de Médecine de Paris. *See* Bulletin de la Faculté de Médecine. Année 1813. No. 8

‡ Essay on Respiration. 8vo. 1804. This has been translated into German by Nolde, under the title of Versuch uber das Athemholen. Mit einem Kupfer, 8vo. Erfurt. 1809.

The diaphragm is a muscle constantly tense, even when quiescent; and the circumference of which terminates, for most part, at moveable points, such as the cartilages of the ribs. As, on contracting, it serves to depress the ribs, so every mechanical movement of these is propagated to the diaphragm. Let the abdomen of an infant be opened, and the contained viscera be removed, in order that the muscular septum, separating it from the thorax, may be more conveniently inspected. Let pressure then be made on the inferior part of the chest, either laterally or in a sterno-vertebral direction, and this pressure be suddenly discontinued; and the costal cartilages, in resuming, from their elastic property, the original state, will be seen to communicate a shock to the diaphragm, continuing as long as the vibrations of the cartilages which have been compressed. It will also be observed, that the more the thorax has been compressed, the stronger will be the re-action of the cartilages; so that the thorax, before returning to its ordinary dimensions, will, for a moment, exceed them, from the operation of those laws which govern the motions of elastic bodies. This experiment, repeated on the adult subject, will yield similar results, except that the shock produced on the diaphragm will be weaker, in consequence of the cartilages of the ribs being much shorter, relatively to the osseous portion, in the adult than in the fœtus. Of the exact researches which I have instituted on this point, I think it right to communicate the outline, especially as nothing of the kind is met with in works of anatomy; and they belong essentially to my subject.

I have measured the length of the seven superior ribs, beginning at the first. The five inferior were omitted, because they do not terminate immediately at the sternum; and, in the region which they occupy, the capacity of the thorax is already diminished.

The following Table* exhibits the results of this admeasurement:

| Cartilage of the 1st rib measures relatively to its osseous portion, as | | 2d Rib | 3d Rib | 4th Rib | 5th Rib | 6th Rib | 7th Rib. |
|---|---|---|---|---|---|---|---|
| In the fœtus . . | 1 : 1, 3 | 1 : 2 | 1 : 3 | 1 : 3 | 2 : 3 | 2 : 3 | 3 : 3,7 |
| —— adult . . | 1 : 4, 6 | 1 : 11 | 2 : 14 | 3 : 14 | 3 : 12 | 6 : 13 | 6 : 13 |

So that, to take the mean measurement for all the ribs in general, one-third of the frame of the chest is cartilaginous in the fœtus, and one-seventh only in the adult. Whence it results, that the parietes of this cavity are incomparably more elastic and compressible in the former than the latter. In these researches, the ribs of the mature fœtus were compared to those of an adult male, aged thirty-three.

We shall terminate this Memoir in our next number. Editors.

* This Table does not exist in the original. We have framed it from the author's materials, for the sake of brevity. Ed.

### II. Pathology. — *Case of large Bronchocele compressing the Trachea.**

J. M. a boy of lymphatic temperament and apparently delicate, had, with the exception of a double inguinal hernia, been free from any morbid affection till his 13th year; at which time he was attacked with measles. The eruption, otherwise regular and judiciously treated, was preceded by slight catarrhal affection with expectoration. Soon after recovery, the respiration of the boy became habitually less free. About the 14th year, the dyspnœa growing constantly worse, the neck was first observed to be enlarged. He gained flesh from the use of asses' milk. Secret remedies were tried, for some months, by an empiric; but the bronchocele and dyspnœa increased. January 4th, 1815, I first saw him. He was then aged 15, and displayed signs of puberty. His figure, of a middle height, shewed nothing remarkable. The symptoms were a large bronchocele, extending laterally; violent palpitations; unequal pulsations of the two radial arteries; respiration extremely difficult, habitually stertorous; all the thoracic and cervical muscles strongly contracted during inspiration, which was performed at times with horrible hissing. No external malformation, except a deep depression just above the ensiform cartilage. No derangement of the digestive, secretory or excretory functions.

A lesion of the heart evidently existed. But what connection had such disease with the disordered respiration? I thought that the trachea was compressed and diminished by the bronchocele. Moving the tumour in different directions, I observed, that when it was pressed laterally, however slightly, respiration was at once suspended. Hence I inferred, that the trachea was laterally flattened, so as to leave but a narrow passage for the air. Two distinct and independent diseases, having no point of relation except the constitution of the patient, therefore, existed. Such a diagnosis led to an unfavourable prognosis. Both I and Dr. Kapeler thought that the ingress of air would at length become completely obstructed by the increase of the bronchocele, and thus the youth perish from asphyxia. The disease of the heart, though far advanced, was less directly menacing. Hence, without any hope of curing, I tried to arrest the progress of the tumour; and, with this view, decided upon the treatment proposed by Godelle.† It was commenced on January the 12th. After recommending the neck to be wrapped in swans-down, I applied a blister to the arm; and, on the 13th, directed a sponge bolus to be swallowed morning and

---

* Observation sur un Goitre volumineux, comprimant la Trachée-artère. Par M. Lullier Winslow, D. M. P. Bulletin de l'Athénée de Médecine de Paris, &c.

† Topographie Médicale de l'Arrondissement de Soissons. Bibliothèque Méd. tome xxxix.

evening.* On the 18th, I prescribed a purgative,† which produced copious evacuations, and seemed to relieve my patient. 21st. Dyspnœa aggravated. 22d. Respiration more sonorous and hissing than ever. Cough came on. Treatment continued; and a linctus, with a grain and a half of kermes (precipitated sulphuret of antimony), occasionally given in addition. During the night of the 23d, my patient suffered extraordinary dyspnœa, and was terrified by optical and acoustic illusions. He called for help; arose, seated himself near the fire, and, feeling better in about an hour, again laid down. Between five and six o'clock, he was found dead, seated on the edge of the bed, and fallen back, with his stockings, slippers, and drawers on. From the position in which he was discovered, and the degree of stiffness, it is probable that he felt uneasy soon after he was left, and made efforts to rise; that the dyspnœa becoming extreme, he lost his balance, in throwing himself back for relief; and that he died from the pressure exercised by the integuments and muscles of the neck upon the tumour, and, consequently, on the trachea.

The following were the results of this important dissection.—*External condition.* No emaciation; some discoloured marks on the limbs, even anteriorly. Thorax little sonorous in the inferior third part on the right side, and about the region of the heart.

*Internally. Thorax.* Sternum less convex internally than concave externally. The thymus gland remaining with all its lobes, and possessing the volume of a duck's egg. Lungs crepitous and sound. The heart large as the two fists of the subject: a white membranous patch seen on the inferior surface of the right ventricle. This cavity was dilated at least one third; and its parietes, especially the anterior, were much extenuated. The auricle was little larger than natural. The left ventricle was dilated. The columnæ carneæ, of the usual size, were less interlaced. The thickness and consistence of the parietes were here not sensibly increased. There was no polypous concretion; no valvular disease.

*Tumour of the neck.* The subcutaneous cellular membrane, surrounding the tumour, was moister than elsewhere. This tumour was formed by the thyroid gland; and extended far on each side, but farthest to the left. The sterno-mastoid and sterno-hyoid muscles were become flat and thin. The tumour, on dissection, presented the natural figure of the thyroid. It was four inches and a half across, by three and a half in the vertical direction, and united to the neighbouring parts by loose cellular substance separable with the finger; but it adhered very intimately to the larynx. The nerves and accompanying arteries were sound. The trachea was imbedded in the tumour. It was flattened laterally, like a

---

* R. Pulv. spong. ustæ dr. j; conserv. cort. aurant. gr. xviij; syr. e rheo q. s. ut f. bolus.

† R. Rad. jalap. pulv.—calomel. aa gr. vj. Misce, s. art.

sabre-sheath, to the extent of an inch and a half, being scarcely, towards the centre of the deformed part, a line broad anteriorly, and a line and a half posteriorly. The superior and inferior portions of the trachea, towards the contracted portion, resembled two funnels, the lower of which was inverted. The voluminous thyroid, detached and incised in several directions, presented no alteration of structure. It weighed about one pound.

The tongue, more firm than natural, presented about thirty papillæ, larger than grains of hemp-seed. The muscles of the base of the tongue, hardened and enlarged, formed above the larynx a tumour of the size of half a small apple. The larynx was small and deformed, so that the rima was found situated to the left, and its right border more elevated than the other. All the abdominal viscera were sound.

---

### III. *Case of Transformation of an Ovary into a scirrhous Mass weighing fifty-five Pounds.**

A woman, aged 24, was imprudent enough, on the seventh day from her confinement, to walk through the snow to a fete at a village distant half a mile, and to return at midnight. Terror, which she experienced on arrival at home, aggravated the operation of the other morbific causes to which she had been exposed. The lochia were suppressed, the mammæ shrunk, and fever came on in succession; followed by severe uterine pains, tension and soreness about the left of the hypogastrium, and numbness of the corresponding thigh. Such was the origin of an organic lesion, which did not prove fatal till after a lapse of twenty-two years. Contrary to the opinion of M. Normand, stimulant treatment was adopted, and the inflammatory symptoms were aggravated by it. The patient seemed, at first, to derive some benefit from recurrence to the antiphlogistic plan; but hectic fever became presently established; the hypogastric pains were renewed; the urine grew scanty; the extremities œdematous; an obscure fluctuation was perceptible in the abdomen. These symptoms, however, yielded to the administration of purgatives, diuretics, resolvents, and tonics; but an induration, as large as a goose-egg, remained in the left hypogastric region. From this time the patient rejected all remedies, with the exception of a caustic.

The tumour remained stationary for two years; when, in consequence of another labour, the symptoms were reproduced. These were successfully combated; but, after their disappearance, the tumour had acquired the volume of an infant's head. Small ulcers formed spontaneously on the legs, which M. Normand thought it

---

* Observation sur la Transformation d'un Ovaire en une Masse squirrheuse du Poids de cinquante-cinq livres, &c. Par Claude Normande, Père, Chirurgien a Courtezols, &c. Recueil periodique de la Société de Médecine de Paris, Mai et Juin, 1816.

imprudent to cicatrize. During the eight succeeding years, the woman twice lay in; and parturition exerted no unfavourable influence on the tumour. But, while M. Normand was absent with the armies, a young surgeon persuaded the patient to heal the caustic, to dry the discharge from the crural ulcerations, and employ pressure, with a view of removiug the swelling of the legs. Soon afterwards, the ovarian tumour became the centre of fluctuation, and acquired, progressively, an enormous volume. The ulcers, which had been so inconsiderately healed, were again opened; but it was now too late. After languishing twelve years longer, towards the close of which she suffered frequent mænorrhagia, the patient sank in extreme marasmus. The following were the principal results of *dissection:* — All the abdominal muscles were completely obliterated. The peritonæum, cartilaginous in a great part of its extent, had acquired, in its middle and inferior portions, a thickness of more than three lines. An enormous, oval, indurated mass, *weighing fifty-five pounds*, filled the whole abdominal cavity. It seemed to be formed by a degeneration of the left ovary. Its substance was composed of membranous cells, more or less thick, filled with a fatty and concrete substance, which, upon incision, several times resisted the edge of the knife.* The other organs presented no considerable lesion.

### IV. Therapeutics—*Neuralgia of the Spermatic Cord, cured by Moxa.*†

A man, aged 35, of a nervous lymphatic temperament, had been subject, from the age of 20, to a slight eruption, consequent on itch, and frequently recurring in winter. At the age of 25, a violent pain of the right temple, returning every morning from ten o'clock till noon, was removed by the application of a blister to the nucha. During his 29th and 30th years, he felt, at times, tearing pains in the left epididymis and spermatic cord. They attracted little attention. He lived very regularly. At the age of 30, the pain became more violent, and considerable testicular inflammation ensued; and was subdued, after three weeks, by leeches, poultices, and diluents. Thenceforth, the pain was uninterrupted, but variable in severity. In the worst paroxysms, commonly brought on by erections and involuntary nocturnal emissions, the pain radiated to the breech, to the left thigh and leg, in the course of the vas deferens, to the basis of the bladder and urethra. It induced frequent desire to void urine, with a sense of scalding. Sometimes,

* None of the characters of *genuine scirrhus* appear to us to have existed in this case. The internal structure, and enormous volume of the morbid ovary, bespeak a widely different affection. Edit.

† Névralgie du Cordon Spermatique, guérie par le Moxa. Observation rapportée à l'Académie de Médecine de Paris; par M. F. Barras, D. M. Gazette de Santé, Septembre 1816.

it became so violent, that the patient lost his appetite, was low spirited, and wished for the extirpation of the testis. The organ was more or less swollen, according to the severity of the pain. A caustic on the thigh was of no service. Leeches, poultices, anodynes in every form; plasters of opium and cicuta, and other topical applications of that class, aggravated the disease. Mercurial frictions, employed under erroneous suspicions of a venereal taint, and the anti-psoric treatment, had a similar effect. Ice, applied to the spermatic cord, produced great but imperfect relief. The pain was diminished by a blister, so long as the cuticular inflammation lasted. A seton was introduced, with the same results. Vinous infusion of valerian, bitters, and antispasmodics, rendered the pain more severe.

The patient then renounced all topical means, except frictions with sulphuric ether, from which he derived momentary relief; and only kept open an issue, and wore a suspensory bandage. The testicle and epididymis retained the natural volume: and, by dint of strict regimen and attention, the pains became tolerable; but they were immediately re-excited by the slightest deviation in regimen. Under these circumstances, moxa was applied on the painful part. The pain, at first, ceased; but recurred slightly on separation of the eschar. A second, applied fifteen days afterwards, again removed the pain; and it was again felt when the slough was detached: yet only by intervals, and withal, so slightly, that the patient paid no farther attention to it.

---

### V.—*New Method of curing Cancerous Ulcers* *

In the different interviews with M. Heschel, Surgeon of the Austrian Military Hospital established at Colmar, he informed me of a successful method which he employs in cancerous ulcers of the face. He assured me that, before his departure from Vienna, he had completely cured by it a man of rank, aged 70, suffering from cancer of the malar region, which was spreading rapidly towards the nose, and had baffled the skill of the ablest surgeons of Vienna. The following is his process:—He touches the scirrhous inverted margins of the ulcer with nitric acid (*acide nitrique fumant*). For this purpose, he employs a golden probe, as all other metals are oxydized by the acid. He passes the probe, moistened with the acid, slightly over the borders of the cancer, and repeats the process two or three days successively. It is discontinued, when the inflammation rises high; and then re-applied till the borders are destroyed by sloughing, and the ulcer is reduced to the state of a simple sore. This invariably happens where the disease is merely local. The dressing consists only of lint moistened in a mixture of water, gum arabic, and brandy.

---

* Nouvelle Méthode pour la guérison des Ulcères cancéreux. Par M. Méglin, Médecin à Colmar. Bulletin de l'Athénée de Médecine de Paris, Mai, 1816.

A short time since, I recommended this process to a man, aged 68, of mucoso-nervous constitution, who had a cancerous ulcer on the lower part of the right leg. Its borders were callous and inverted; the sanies, discharged from it, excessively fœtid, and the attendant pains unusually severe. The ulcer spread in all directions, notwithstanding the efforts of an experienced surgeon. The nitric acid, employed on the plan of M. Heschel, caused the scirrhous borders to slough. The wound, rendered simple, was merely dressed with adhesive straps, according to the English method, of late and most deservedly eulogized in France; and the cancerous ulcer is, at this moment, completely healed. The plan of M. Heschel is certainly entitled to the attention of professional men.

---

VI. *Medical Jurisprudence.* Article, *Blessure*, *Wound;* Gr. τραυμα; Lat. *Vulnus;* Germ. *Wunde;* It. *Ferita.*—Considered in its relations with juridical medicine.

Examination of the effects produced upon one or more of our organs by external violence constitutes, in juridical medicine, the doctrine of wounds. By the term wound, solution of continuity is not invariably implied; although some authors exclusively restrict it to the sense in which it is used in common language. Perhaps, it might be well to substitute, as a generic term, *lesion from an external cause*, for *wound*, in juridical language; as being capable of application more general and precise.

There are two modes of contemplating and dividing lesions, in Forensic medicine. The one, essentially anatomical, is determined by their situation: the other, on the contrary, is juridical, and rather regards the consequences of such lesions to the life and health of the individual wounded. The attention of physicians and legislators has been particularly occupied by the latter of these divisions, since the criminal code of the Emperor, Charles V. in some degree proportioned the punishment to the physical effect of crimes. The existing penal laws of France (and of England,* we may add) which decide upon and punish, rather the intention than the effects, seem, at first sight, to render useless these distinctions. Nevertheless, there are many dispositions, both in the civil and criminal codes which necessarily require them. Again, some division of lesions, according to their situation and mortality, is equally essential for the instruction of the jury and the council. Consideration of the instrument with which the wound has been inflicted and of the degree of violence employed, will frequently throw light upon the intention of the prisoner, as indicated by his choice of means. Now cases may occur in which death has no obvious connection with the cause supposed to have determined it; and they, consequently, require the most

---

* Particularly since the promulgation of the celebrated Ellenborough Act.

deliberate investigation of the individual circumstances capable of inducing results thus unexpected. An individual is stricken on the head and dies: and can it be a matter of indifference to the judge and to the prisoner's counsel whether the injury have been inflicted with a switch or an iron bar. Now, supposing the former to have been the fatal weapon, and that it have struck a part of the occipital bone, nearly destroyed by caries, ought not the fatal event to be wholly attributed to this unfortunate peculiarity? And can we rationally suspect that the conduct of the prisoner was actuated by what is termed, in the language of the law, the *animus occidendi?*

Every one, however little he may have advanced in the study of the phænomena of life, will readily descry the difficulties by which a systematic division of lesions, according to the degree of their mortality, is opposed. "The countless varieties," says M. Foderé, "which Nature exhibits, render all methods in some point of view defective. Common wounds may acquire, from some adventitious cause, a dangerous character. A slight blow, inflicted on the leg of an unhealthy subject, is often followed by consequences which call for amputation. From a trivial incision of the finger, we have seen gangrene invade the hand and arm. A trifling contusion on the breast of a woman, disposed to cancer, may induce dreadful mischief. Again, in the army we have witnessed the most miraculous recoveries from wounds penetrating and injuring the more important viscera."* Here, as elsewhere, we should prefer the method which is simplest and most conformable to the duties required from the physician in a court of justice.

Lesions are mortal or they are not: the latter may be distinguished as perfectly or imperfectly curable. Of these the last are appreciated, from the importance of the function injured and the incapacity, consequent on the wound, to execute the ordinary duties of life. The slightness of some wounds allows us to pronounce them completely curable ere the prognosis can be confirmed by the event. Others, again, can only be so declared after a protracted treatment.

To determine the mortality of a wound, it is not necessary that death should directly, or very soon follow it; provided that the event is proved to be the consequence of the violence inflicted. Yet would it be an error in every case to regard the death, speedily consequent on a wound, as determined by the injury. A scuffle, for instance, takes place between two persons. One of them, of a constitution decidedly apoplectic, receives from his antagonist a slight blow on the head or elsewhere, and instantly expires; not in consequence of the external lesion, but of apoplexy determined by rage. In other cases, death may be justly attributed to the injury; although not occurring for weeks or even months after its infliction. This frequently is observed in le-

---

* Traité de Médecine légale, &c. (*First Edition.*)

sions of the breast, inducing hæmoptoe or phthisis. Such considerations have not always sufficiently influenced legislators, in their attempts to restrict within certain periods the mortality of wounds, and to establish differences in this respect, which are utterly inconsistent with daily observation, and which no rational physician will hesitate to reject.

( To be continued. )

---

